# Health and Gender Inequalities of the COVID-19 Pandemic: Adverse Impacts on Women's Health, Wealth and Social Welfare

**DOI:** 10.3389/fgwh.2021.670310

**Published:** 2021-07-20

**Authors:** Roberta Guerrina, Bettina Borisch, Leigh F. Callahan, Jeremy Howick, Jean-Yves Reginster, Ali Mobasheri

**Affiliations:** ^1^School of Sociology, Politics and International Studies, University of Bristol, Bristol, United Kingdom; ^2^Policies and Governance Research Group, Institute of Global Health, University of Geneva, Geneva, Switzerland; ^3^Thurston Arthritis Research Center, School of Medicine, University of North Carolina, Chapel Hill, NC, United States; ^4^Division of Rheumatology, Allergy and Immunology, Department of Medicine, University of North Carolina, Chapel Hill, NC, United States; ^5^Departments of Orthopedics and Social Medicine, University of North Carolina, Chapel Hill, NC, United States; ^6^Faculty of Philosophy, University of Oxford, Oxford, United Kingdom; ^7^World Health Organization Collaborating Centre for Public Health Aspects of Musculoskeletal Health and Aging, Liege, Belgium; ^8^Research Unit of Medical Imaging, Physics and Technology, Faculty of Medicine, University of Oulu, Oulu, Finland; ^9^Department of Regenerative Medicine, State Research Institute Centre for Innovative Medicine, Vilnius, Lithuania; ^10^Departments of Orthopedics, Rheumatology and Clinical Immunology, University Medical Center Utrecht, Utrecht, Netherlands; ^11^Department of Joint Surgery, First Affiliated Hospital of Sun Yat-sen University, Guangzhou, China

**Keywords:** COVID-19, pandemic, gender, global health, women's health

## Abstract

In this paper we discuss the nexus of health and gender inequalities associated with the COVID-19 pandemic and highlight its adverse impacts on women's health, welfare and social standing. The COVID-19 pandemic has exposed the link between socio-economic inequalities and health outcomes, especially in the area of rheumatic and musculoskeletal (RMDs) diseases. Women are more adversely affected by RMDs diseases compared to men. Epidemiological research carried out over several decades has demonstrated the presence of clear gender patterns in the manifestation of musculoskeletal diseases, including osteoarthritis (OA), rheumatoid arthritis (RA), systemic lupus erythematosus (SLE), systemic sclerosis (SS) and osteoporosis (OP). The public health measures that have been adopted to curb the spread of Sars-COV-2 are expected to have a particularly detrimental impact on women in the long term precisely because of the nexus between health outcomes and socio-economic structures. Moreover, the prioritization of urgent care will further compound this effect. COVID-19 has created a condition of ontological insecurity that is becoming increasingly manifested through various chronic diseases and associated comorbidities. RMDs and their impact on mobility and the ability of individuals to be independent, happy and mobile is a key public health challenge in the post-COVID-19 reality and a key part of the ongoing pandemic. There is an urgent need to engage with policymakers to publicize and prioritize this problem and develop viable solutions to address it.

## Introduction

Coronavirus disease (COVID-19) is an infectious disease caused by a newly discovered coronavirus (1). Most people infected with the COVID-19 virus experience mild to moderate respiratory illness and recover without requiring special treatment (2). However, older individuals, and those with underlying medical problems like cardiovascular disease, diabetes, chronic respiratory disease, and cancer are more likely to develop serious illness and die from COVID-19 (3). At the time of writing (20 February 2021) this pandemic has resulted in more than 2,467,342 deaths according to the World Health Organization^1^ and the European Center for Disease Prevention and Control^2^.

The COVID-19 pandemic has had unprecedented and potentially irreversible impacts on health and healthcare globally with ongoing and adverse impacts on the economy (4). There are significant health, race and gender inequalities associated with the COVID-19 pandemic (5). There are therefore many complex issues and factors that need to be accounted for as we look at the long-term impact of COVID-19 on the very fabric of humanity and society and how this ongoing crisis continues to affect health and social care outcomes for different groups. This paper aims to summarize our key concerns in relation to the social determinants of health (SDH).

**Figure 1:**
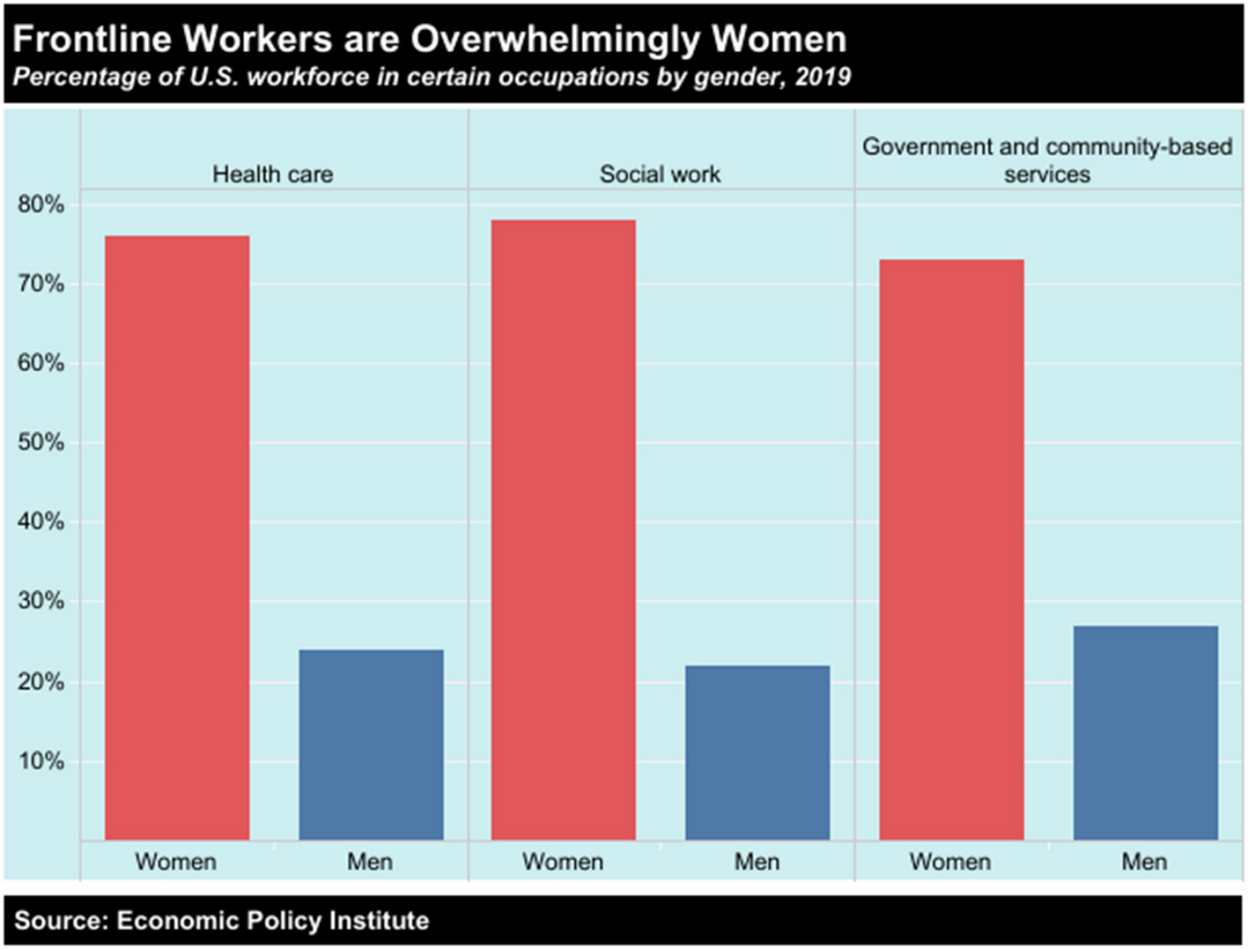
Frontline jobs, which are deemed “essential” and require people to work in-person, are heavily staffed by women. The healthcare, social work, and government and community-based services sectors are overwhelmingly made up of female employees, according to research from the Economic Policy Institute (https://www.epi.org/).

**Figure 2:**
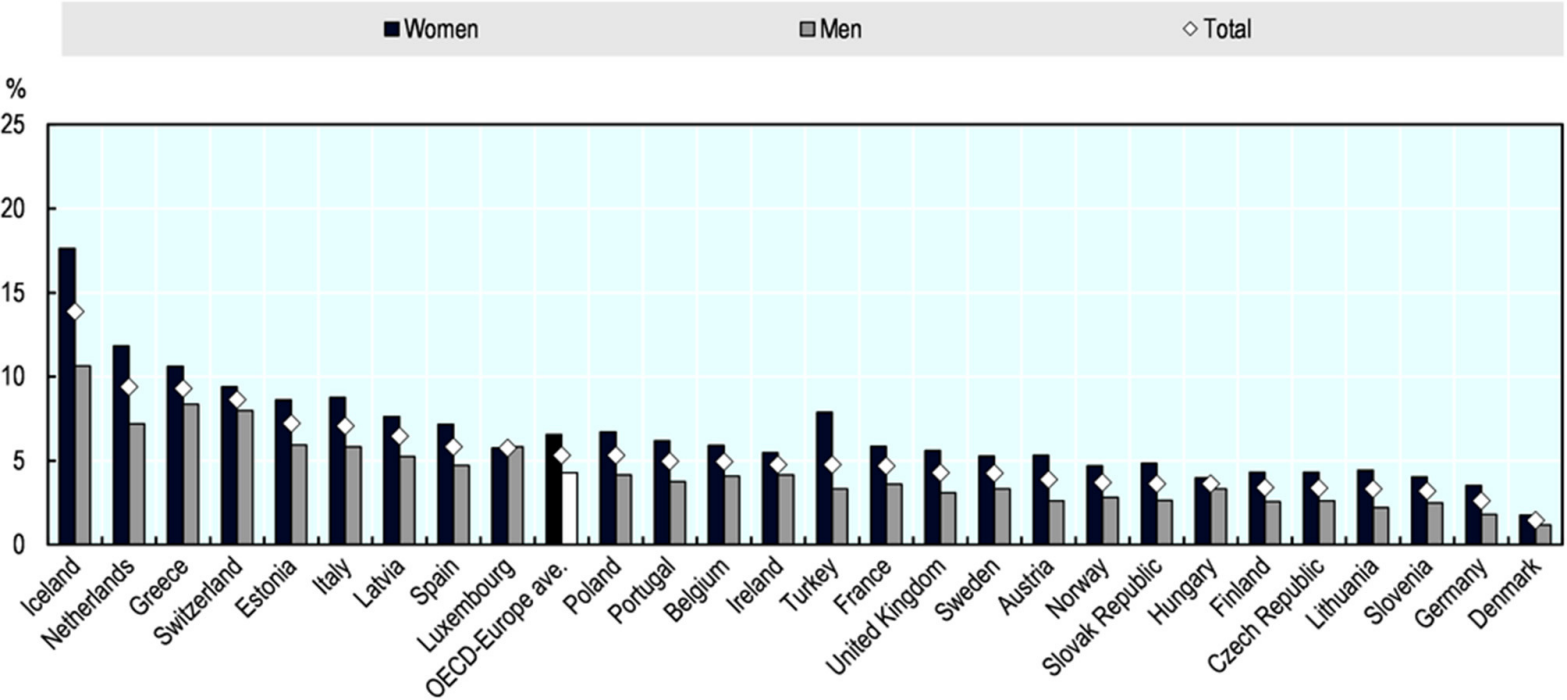
Employed women are much more likely than employed men to have care responsibilities. Share of employed persons reporting that they regularly take care of ill or disabled or elderly adult relatives, by sex, European OECD countries, 2018. The data represents share of the employed population who report taking care of ill or disabled or elderly adult relatives (15-year-olds and older), regularly. The relative may live in- or outside the household. Source: Eurostat Database, based on the European Union Labor Force Survey *ad-hoc* module 2018, https://ec.europa.eu/eurostat/data/database.

A very brief analysis of both gender and race in the context of the global pandemic highlight a number of issues that warrant further consideration:

- Frontline workers are overwhelmingly women (Figure 1) and employed women are much more likely than employed men to have care responsibilities (Figure 2). The high proportion of women deployed as “frontline” service workers across a range of professions (6). This arises from both the vertical and the horizontal segregation of the labor market. In other words, women tend to occupy lower paid positions and are often associated with social function of “care.” These same positions have historically been classed as “low skilled” to justify lower pay in care services and delivery. Women thus play a disproportionate role in frontline health and social care roles and perform the majority of caregiving responsibilities.
- Growing awareness of the role of key workers during this latest crisis could provide a moment of reflection and recognition about the centrality of these roles to society and the economy. It *should* thus open a space to revalue “care” as a function that is central to the human condition; such reckoning or recognition, however, will require the political ambition to imagine a post-COVID-19 recovery in which care is an integral part of the economic infrastructure.
- Gender disparities are also emerging in terms of health outcomes. As a result gendered work and division of the healthcare labor market, women are more exposed to COVID-19, and at a much higher viral load than men (6). We do not yet know the long term health consequences of this level of exposure. Whereas, women make up a smaller percentage of the severe COVID-19 cases presenting in hospitals (7), they seem to be more likely to suffer from long-COVID-19 (8).
- There are also serious issues regarding the impact of COVID-19 on Black, Asian and Minority Ethnic (BAME) communities. A systematic review of the published literature on COVID-19 articles in some of the most prestigious medical journals including New England Journal of Medicine, Lancet, British Medical Journal and the Journal of the American Medical Association plus EMBASE, MEDLINE, Cochrane Library, PROSPERO, clinical trial protocols, gray literature, surveillance data and preprint articles in MedRxiv has revealed that BAME individuals had an increased risk of infection with SARS-CoV-2 compared to white individuals and, 12 studies eported worse clinical outcomes, including intensive care unit admission and mortality (9). It is interesting to note that traditionally, very few analyses have drawn the link between co-morbidities associated with pandemics and socio-economic structures e.g., gender, class and race. The wealth of data collected during this pandemic is forcing us to re-evaluate the way we think about the serious shortcoming of any socio-political, economic and medical analysis of the pandemic that does not centers the link between these “social issues” and health outcomes.
- COVID-19 is bringing to light a number of “blind-spots” in public and health policy (10). It is thus an opportunity to draw attention to the importance of impact assessments, not just in policy making but also research in order to avoid “unintended” consequences that have an asymmetrical impact on different demographic groups.

This paper is the result of a series of e-mail exchanges, Zoom meetings, telephone conversations and videoconferences between a group of scientists working in different disciplines including medicine, political sciences, global health and philosophy. Three of the authors have interest and expertise in the field of musculoskeletal diseases. Therefore, an inevitable consequence of this collaboration is the framing of some of the adverse impacts of the COVID-19 pandemic in the context of mobility, or rather reduced physical activity during the lockdown phases of the pandemic and musculoskeletal health and disease in women. However, before focusing on musculoskeletal health in women we explore the concept of gender imbalance in the aftermath of the COVID-19 pandemic.

## Gendering COVID-19

Mapping the multiple and intersecting ways that governments respond to COVID-19 affects different demographic groups in the short, medium and long term is crucial for understanding the immediate and long-term socio-economic impacts of the 2020 pandemic (11–15). There is a wealth of evidence that directly links health outcomes to socio-economic factors. Poverty, poor education, tobacco use, unhealthy diets, physical inactivity and excessive consumption of alcohol are factors that determine poor health outcomes (16). Often cited predictors of positive health outcomes are a nutritional balanced diet and free access to safe places for engaging in physical activity (17–20). Perhaps focusing on diet and exercise is not surprising as they can be easily quantified and correlated to the onset of disease, as well as its course of progression. However, there are also a number of “soft” factors that intervene and exacerbate the impact of economic and environmental factors on health outcomes. These soft factors, e.g., gender divisions of labor in the domestic sphere or cultural factors/norms determine opportunities and constraints for different groups of the population to adhere to health advice. When trying to understand the equality and diversity impact of government responses to COVID-19 it is essential that population groups are not treated as homogenous. Populations are extremely heterogeneous at all levels of society and it is crucial for the society to be viewed as multi-layered and diverse. For instance the experience of women during the various COVID-19 “lockdowns” and quarantines will be determined by their socio-economic status, their religious and cultural affiliations, education, ethnic background, employment and parental status, specifically the age of their children.

The focus of our analysis revolves around the “unintended consequences” of policy decisions that fail to carry out adequate equality and diversity impact assessment at the point in which policies are being designed and decided. There is a note of caution here: the nature of the current crisis created an inevitable urgency to act or be seen to act. It also required policy makers to identify priorities, including health priorities. This is not in itself a problem, however lack of representation of diverse sets of interests at the conceptualization stages of any policy are likely to entrench implicit bias by overlooking the concerns of minoritized and marginalized groups.

In terms of gendered dynamics a few initial observations can give us pause for thought. First and foremost, quarantine is predicated on the assumption that the “threat” to an individual's health and safety occurs in the public sphere. Hence the slogan: “stay home, save lives…” Another important aspect is the “individualization of experience,” primarily lived inside one's home, is a building block of civic responsibility … “we are in it together.” This discursive framing, however, disregards the fact that the domestic sphere is not a place of safety for survivors of domestic abuse. Hence the increase in calls to helplines which speaks to current work on (gendered) regimes of violence (21).

It is in this context that important dynamics can be observed that highlight the continued impact of gender regimes in women's experience of work-life balance. Aside from gender differences in patients with COVID-19 (22), recent surveys are pointing to the reassertion of private gender regime as a result of the pandemic. Social reproduction increases women's responsibilities to provide care, in many cases in addition to paid work. Quarantine decisions have had a direct impact on women's freedom and their ability to exercise and adhere to medical advice. For instance, school closures and requirements for home schooling, particularly of primary age children is having an impact on women's earnings and their financial independence. As a result of the struggle to balance paid work and social reproduction many women are likely to be affected in the short, medium and long term by the decisions taken in the first half of 2020 and, in the UK and many other countries in lockdowns introduced as the second wave was starting. These patterns have, in turn, had a detrimental impact on women's access to “leisure” including exercise required for managing musculoskeletal conditions, which we will focus on.

Women make up the majority of the health and social care workforce on the “front line” of the COVID-19 pandemic, e.g., as nurses and carers (23–25). It is interesting to note that the feminisation of the labor force and economic activity in the public sphere during the pandemic also reflects women's nurturing role in social reproduction. Quarantine requires individuals to take responsibility for their health by decreasing movement and adhering to strict confinement guidelines. In this context, “failure” to continue with exercise regimes and physical activity to manage health conditions is the responsibility of an individual and in some countries and territories it is a shared decision-making process with a healthcare practitioner.

This has allowed policy-makers to overlook the impact of women's role in social reproduction and the increasing weight of the double burden in the domestic sphere. Quarantine is also prioritizing COVID-19 as a health “threat.” This approach reinforces the health pyramid, which positions musculoskeletal health much lower on list of priorities for healthcare providers. It is well-established that a range of chronic conditions that affect minoritized and marginal groups are often overlooked. The question to address here is whether COVID-19 will compound existing problems of unconscious bias in medicine, and how these interact with wider gender norms and hierarchies during times of crisis.

Here are some key questions from an Equality, Diversity and Inclusion (EDI) perspective:

- What are the pathways for incorporating concerns around equality and inclusion into the story of COVID-19? There is substantial discussion at the moment about highly visible issues, e.g., higher death rates amongst black and minority communities, domestic abuse, but will this be included in the official record of the 2020 pandemic?
- What are the “blind spots” of policy? For example the impact of gender divisions of labor and the double burden on women's experience of public health measures during the Covid-19 crisis. What is the short, medium and long term impact of such omissions?
- How will the policy decisions of the first 6 months of 2020 affect men and women's experiences and health outcomes in the next 5 or 10 years?
- What are the costs of failing to carry out equality and diversity impact assessments in the context of this crisis?

These questions need to be examined within a broad context that positions the equalities agenda at the heart of the national and transnational policy agenda. The Sustainable Development Goals specifically include obligations to advance gender equality (SDG5) and reducing inequalities (SDG10).

## The International Context: The Sustainable Development Goals 5 and 10

A total of 17 Sustainable Development Goals (SDG) were established by the United Nations in 2015^3^. Sustainable Development Goals 5 and 10 focusing, respectively, on gender equality and reduced inequalities are particularly relevant to this paper. The official wording of SDG 5 is “Achieve gender equality and empower all women and girls.” The official wording of SDG 10 is “Reduce inequality within and among countries” (26).

With regard to SDG 10 and COVID-19, there are two area to focus on, especially in relation to health outcomes:

- It is important not to assume that women's experience of COVID is homogeneous. Although a lot of the focus of the media coverage is on reconciliation between work and family life experienced by mostly white professional women, who became responsible for home schooling etc., the challenges faced by many BAME or minoritised women, often employed as frontline workers, is significantly different.
- The international picture is becoming increasingly complex, though there are some trends that can be identified. Intersectionality it's becoming more important and in relation to SDGs and the international trajectory of the COVID-19 pandemic, what is not yet clear is what role are women playing in the global south in managing community based responses to the pandemic.

The SDG implicitly and explicitly recognize that development, cohesion, and social justice go hand in hand. COVID-19, however, expose the varied ways in which socio-economic inequalities shape public health approaches and thus defined health outcomes. In the next two sections we will explore the interaction between the social and the physical generates a deep sense of ontological insecurity (27, 28).

## Exacerbation of Musculoskeletal Health Inequalities

Women have a higher prevalence of RMDs including osteoarthritis (OA), rheumatoid arthritis (RA), systemic lupus erythematosus (SLE), systemic sclerosis (SS) and osteoporosis (OP) and probably sarcopenia as well. Therefore, as a consequence women have a much higher risk of developing cardiovascular comorbidities (29–34). Subsequently, any external threats, such as the restrictions and lockdowns experienced during the COVID-19 pandemic, are clearly going to have adverse effects on musculoskeletal disease diagnosis and management and this will affect females more significantly than males (35).

### Osteoporosis, Fractures and Bone Health

The endogenous production of vitamin D is dependent on sunshine exposure (36). However, because of lockdowns and containment, particularly at the end of the Winter and during the Spring, many elderly subjects, especially those in care homes will remain with circulating levels of vitamin D which are insufficient for bone, joint and muscle health, hence increasing their risk for falls and fractures (37).

In many low income countries musculoskeletal diseases are not considered to be health priorities and when epidemics and pandemics occur low income countries take the brunt and bear the harshest consequences (38–40). Even in the global North musculoskeletal disease has had to take second place to the health emergency associated with the pandemic. The pandemic has crystallized a heath hierarchy that prioritizes life threatening conditions over chronic disease. Visits to general practitioners have been significantly hampered during the COVID-19 lockdown (41, 42). Routine and planned diagnostic procedures such as radiography, ultrasound, and magnetic resonance imaging (MRI) have been delayed and postponed. Subsequently, the initiation of treatment for many musculoskeletal diseases has been delayed for many weeks and months with adverse effects on patients who desperately needed it; many of these patients are women. There is evidence from the most advanced economies that orthopedic surgery is now several months behind schedule. It will be extremely difficult, if not almost impossible to catch up. As a result many patients will not receive the surgical care that had been planned for them before the outbreak.

There is now an acute and almost frightening problem in OP management where patients with an incident fracture (spinal, forearm) which does not require hospitalization have had their post-fracture visit postponed until the re-opening of non-urgent consultations/technical examinations (e.g., DXA, X-Rays). Knowing that a recent fracture is one of the major risk factors to present with a new subsequent fracture, this may generate a major risk for these patients.

### Monitoring Treatment

One of the major issues in the management of chronic silent disorders is the lack of adherence to treatment goals. The lockdown has prevented patients from getting their prescriptions renewed on time. Also, many patients have gone without consultations with their general practitioner, which means that they have not received the positive feedback that is normally provided by consultation with their regular physician, hence increasing the risk of premature treatment discontinuation. Some of the drugs (IV formulations) are given under the responsibility of a registered nurse or of a physician. All these treatments were delayed during the lockdown with long-term adverse impacts on women's health.

### Mental Health and Anxiety

Depression and anxiety and physical violence will occur, particularly in people who are contained in small apartments, with children and/or pets. In this case, most of the house work will frequently be the responsibility of the homemaker, frequently women. This additional burden, leading to some kind of domestic burn-out will impact also on the motivation to either take the needed medications, to take physical exercise or to comply with the principles of bone/joint/muscle-health and nutrition. Compliance and adherence often requires input from healthcare practitioners. The lockdown has meant reduced opportunities for interactions between patients and their healthcare practitioners, exacerbating the anxiety associated with disease and the burden of the disease itself.

## COVID-19 as a Juncture for Socio-Economic Inequalities in Musculoskeletal Health Outcomes

As outlined previously COVID-19 has laid bare long standing socio-economic inequalities (5, 43) and wealth inequalities. Beyond the increase in women's double burdened due to remote learning and childcare closures, there are important links to be made between these broader trends and long-term health outcomes for women. Rising levels of gender based violence and abuse have received significant attention, however there are also needs to be research on the impact of public health measures on women's long-term musculoskeletal health, especially relating to OP and OA.

**Figure 3:**
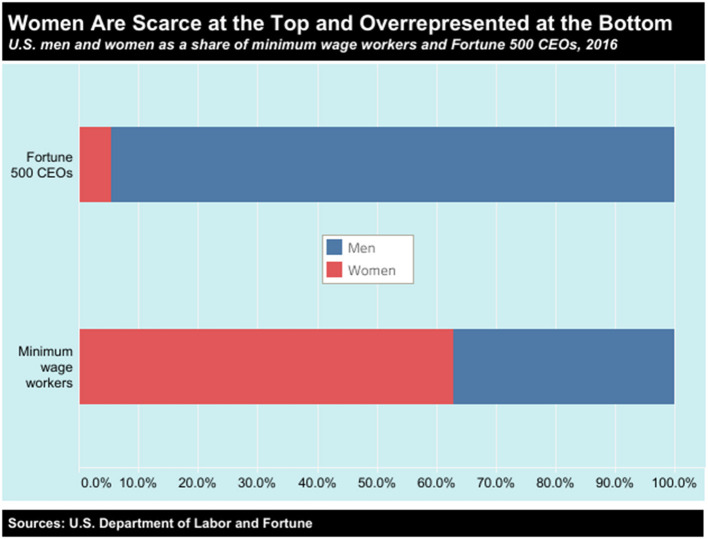
Women are under represented at the top of the wealth pyramid and over represented at the bottom, occupying the lowest paid jobs in society, according to the United States Department of Labor (https://www.dol.gov).

There are important parallels between economic and medical hierarchies. Female-dominated occupations—such as childcare and hospitality—continue to occupy the lower rungs of the wage ladder. In the United States women make up 63 percent of workers earning the federal minimum wage, a wage rate that has been stuck at $7.25 since 2009. In contrast, women represent only 5 percent of CEOs at Fortune 500 firms (Figure 3). The loss of income resulting from public health measures associated with COVID-19 will exacerbate social inequalities in access to healthcare. Musculoskeletal diseases are also located lower in the hierarchy of needs and individuals relying on national health provisions to access treatment for musculoskeletal diseases are likely to be one of the worst affected. Delay in treatment however will not only have an impact on their health outcomes, but it will also curtail their ability to participate fully in the employment market. This will in turn increase the medium and long terms social and economic costs to the individual and society.

Throughout the pandemic political leaders have claimed to “follow the science” (44). The explicit implication of this statement is that science determines a particular policy approach has damaged the relationship between science, society and the development of and implementation of policy (45). Decision making processes, however, are much more complex as scientific evidence is balanced against, or perhaps alongside, wider political priorities. What COVID-19 has highlighted is that it is politics, rather than science, that determines policy. The inference from science to policy is not straightforward and is often highly problematic. If it were, there would not be the wide range of policies being implemented within and across countries. While it is disputed, the Scottish philosopher David Hume warned us against inferring an *ought* from an *is*. Leaving aside the question of whether the science tells us what is (the facts about COVID-19, at least initially, were murky at best), it does not imply any particular course of action. For example, we have known for several months now that COVID-19 is virulent and dangerous, but it does not represent the existential risk that some feared initially. Determining the right policy based on those facts depends on what the harms of the policy are, and what the individual and societal values are. Here are just a few of the considerations that need to be taken into account when “following the science”:

- The economic cost of losing one's job are greater in countries without strong social networks.
- Severe lockdowns cause harm to those without strong social networks, especially individuals who lived alone, and those in isolated communities.
- As mentioned previously, women and BAME people are disproportionately affected.
- The long-term economic damage caused by COVID-19 induced lockdowns will cause more indirect deaths by starvation than deaths caused by the virus according to the United Nations.

Taking these considerations into account doesn't make choosing the right policy any easier. But if we willfully ignore them by continuing to hold on to the mistaken belief that science determines policy, we have no chance of addressing the important issues, which are clearly beyond the scope of this paper.

## Conclusions

In April 2020 the United Nations (UN) published a policy brief on the impact of COVID-19 on girls and women^4^. Although the policy brief was published fairly a few weeks into the start of the pandemic, it did highlight three important priority areas:

- Ensuring women's equal representation in all COVID-19 response planning and decision-making.
- Driving transformative change for equality by addressing the care economy, both paid and unpaid.
- Targeting women and girls in all efforts to address the socio-economic impact of COVID-19.

Almost a year later, it is clear that we have a lot of work left to do. As EU countries re-enter lockdowns during the second wave of COVID-19, governments must learn important lessons from the positive actions taken during the first wave of the pandemic and reflect on the shortcomings of their inevitably reactive approaches during the first wave. In the majority of EU countries, the pandemic has exposed overall shaky support systems for the most vulnerable in society. The crisis continues and despite emerging vaccines and implementation of mass vaccination programmes in 2021, COVID-19 has transformed the way we live and work and made us question our relationships with each other and with our governments. The pandemic has shone a harsh light on how unprepared we have been and highlights the anxieties and uncertainties that will follow us into 2021 and beyond. There is emerging consensus that a better understanding of public perceptions of government responses to COVID-19 may foster improved public cooperation (46). We must work hard to ensure that future efforts emphasize the gender dimension in all possible ways. We must also engage with policymakers to publicize and prioritize this problem and develop viable solutions to address it.

## Data Availability Statement

The original contributions presented in the study are included in the article/supplementary material, further inquiries can be directed to the corresponding author/s.

## Author Contributions

AM conceived the paper, produced the first draft, and edited the manuscript. All authors contributed to the article and approved the submitted version.

## Conflict of Interest

The authors declare that the research was conducted in the absence of any commercial or financial relationships that could be construed as a potential conflict of interest.

## Footnotes

^1^ https://www.who.int

^2^ https://www.ecdc.europa.eu/en/covid-19-pandemic

^3^ https://www.un.org/sustainabledevelopment/blog/2015/12/sustainable-development-goals-kick-off-with-start-of-new-year/

^4^ https://www.un.org/sexualviolenceinconflict/wp-content/uploads/2020/06/report/policy-brief-the-impact-of-covid-19-on-women/policy-brief-the-impact-of-covid-19-on-women-en-1.pd
